# Characterization of an Injury Induced Population of Muscle-Derived Stem Cell-Like Cells

**DOI:** 10.1038/srep17355

**Published:** 2015-11-27

**Authors:** Kinga Vojnits, HaiYing Pan, Xiaodong Mu, Yong Li

**Affiliations:** 1 Department of Pediatric Surgery, University of Texas Medical School at Houston, TX 77030, USA; 2 Center for Stem Cell and Regenerative Medicine, University of Texas Health Science Center at Houston (UTHealth), TX 77030, USA; 3 Stem Cell Research Center, University of Pittsburgh, Medical School, Pittsburgh, PA 15213, USA

## Abstract

We recently discovered a novel population of stem cells from the injured murine skeletal muscle. These injury induced muscle-derived stem cell-like cells (iMuSCs) are partially reprogrammed from differentiated myogenic cells and display a pluripotent-like state. The iMuSCs exhibit stem cell properties including the ability to differentiate into multiple lineages, such as neurogenic and myogenic differentiations; they also display a superior migration capacity that demonstrating a strong ability of muscle engraftment *in vivo*. IMuSCs express several pluripotent and myogenic stem cell markers; have the capability to form embryoid bodies and teratomas, and can differentiate into all three germ layers. Moreover, blastocyst microinjection showed that the iMuSCs contributed to chimeric embryos but could not complete germline transmission. Our results indicate that the iMuSCs are in a partially reprogrammed state of pluripotency, which are generated by the microenvironment of injured skeletal muscle.

Tissue repair after injury is a complex biological process, which involves the activation of tissue-resident precursors and stem cells, and a variety of infiltrating cells responding to local and systemic signals. Mammalian skeletal muscle regeneration relies on the activation and proliferation of the resident muscle precursor cells^1^,^2^ including satellite cells and muscle stem cells (MuSCs), which are populations of mononucleated cells located between the basal lamina and sarcolemma of muscle fibers. MuSCs are a functionally heterogeneous population of cells and have variable proliferation rates, marker expression profiles, self-renewal capacities, clonogenicity and differentiation capacities^2^,^3^. We have previously discovered that among MuSCs, a small population of iMuSCs exist and can be isolated from injured murine skeletal muscles using a Cre-LoxP system established in our laboratory^4^. We have shown that iMuSCs not only express CD34, Sca1 (Stem cell antigen-1), and Pax7 (Paired box protein 7), but also presented strong myogenic differentiation and muscle regeneration abilities *in vivo*^5^. In addition, we demonstrated that iMuSCs demonstrate stem cell behaviors, and are capable of differentiating into non-myogenic lineages, such as CD31^+^ endothelial-like cells in the healed skeletal muscle^4^. Here, we further investigate the unique nature of the iMuSCs, focusing on their morphology, marker expression profile, pluripotency, migratory abilities and differentiation potential.

## Results

**Figure 1:**
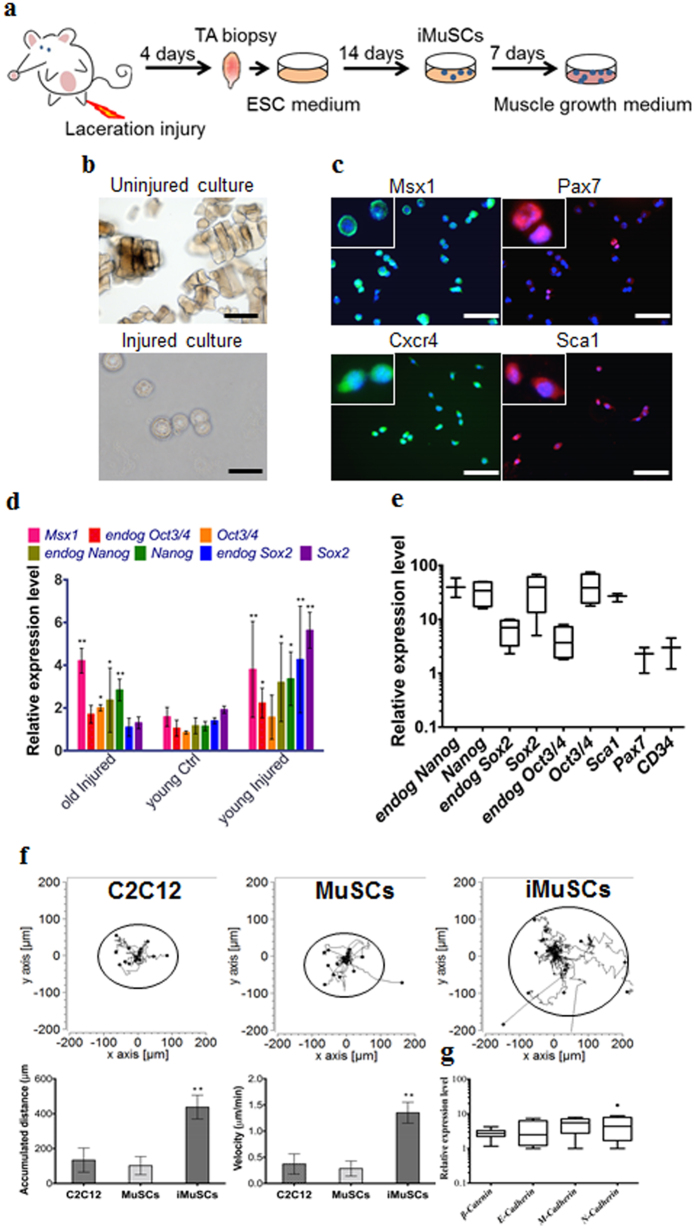
IMuSCs display stemness and exhibit improved migration ability. (**a**) Schematic of iMuSCs isolation method from injured murine TA muscles. (**b**) Bright field images of uninjured and injured cultures. 3 days after the cell isolation no cells appeared in the control uninjured cultures, but iMuSCs were present in the injured cultures. 7 days after cell isolation, the proliferation of iMuSCs was apparent. Scale bar = 10 μm. (**c**) Msx1 (green), Pax7 (red), Cxcr4 (green), and Sca1 (red) expression of iMuSCs. Nuclei were stained with DAPI (blue). Scale bar = 100 μm. (**d**) qPCR analysis of whole biopsied TA muscles, and (**e**), fresh isolated iMuSCs. (**f**) Single cell migration pathways of iMuSCs, and the control C2C12 and MuSCs. The migration paths of 20 individual cells from different experimental groups captured in a time-lapse motility assay. Data was pooled from 3 independent experiments. Graphs show the calculated accumulated distance and velocity of the cells. Data are represented as the mean ±SEM of 60 individual cells from 3 biological replicates. **P < 0.01. (**g**) qPCR analysis of β-Catenin, E-Cadherin, M-Cadherin, N-Cadherin expression of iMuSCs. Data are represented as the mean ±SEM of 5 biological replicates.

By applying our established cell isolation method (Fig. 1a) iMuSCs were successfully isolated from the injured murine tibialis anterior (TA) muscle. Three days after the cell isolation, proliferating iMuSCs (~0.1% of the entire muscle cell population) appeared in the culture dishes; however, no cells were present in the cultures that established from the control, uninjured muscles (Fig. 1b). Microscopic evaluation revealed that representative iMuSCs were 5–7 μm in diameter, contained relatively large nuclei and a narrow rim of cytoplasm. Their nuclei were Hoechst 33342 positive and incorporated BrdU (Bromodeoxyuridine) with Msx1 (Msh homeobox 1) expression (Supplementary Fig. S1a). The freshly isolated, or early population of iMuSCs contained a high percentage of Msx1 and Cxcr4 (C-X-C chemokine receptor type 4) positive cells, among which were a few cells expressing Pax7 and Sca1 (Fig. 1c). Gene expression analysis of the entire biopsied injured TA muscles showed that Msx1, Oct4 (also called Pou5f1), Sox2 (SRY-box 2) and Nanog expression were up-regulated compared to the control uninjured old TA muscle (Fig. 1d and Supplementary Fig. S1b). Freshly isolated iMuSCs expressed myogenic stem cell related markers, i.e. Sca1, Pax7 and CD34, and the core pluripotency marker genes, i.e. Oct4, Sox2 and Nanog (Fig. 1e and Supplementary Fig. S1c). Cultured iMuSCs were expanded *in vitro* in muscle growth medium with an average cell population doubling time of 13 hours. Cytogenetic analysis revealed that iMuSCs had a normal female karyotype; however, chromosomal aberrations appeared during long-term culture (passage 33), resulting in trisomy for chromosome 5 (Supplementary Fig. S1d). We also discovered that iMuSCs had remarkable migration properties. Data from a time-lapse motility assay confirmed that iMuSCs migrated longer distances with higher velocities compared to the control mouse myoblast cell line, C2C12, and MuSCs isolated from control uninjured muscle (Fig. 1f). Moreover, iMuSCs expressed high levels of *β-Catenin* and several *Cadherins* at the mRNA level (Fig. 1g).

**Figure 2:**
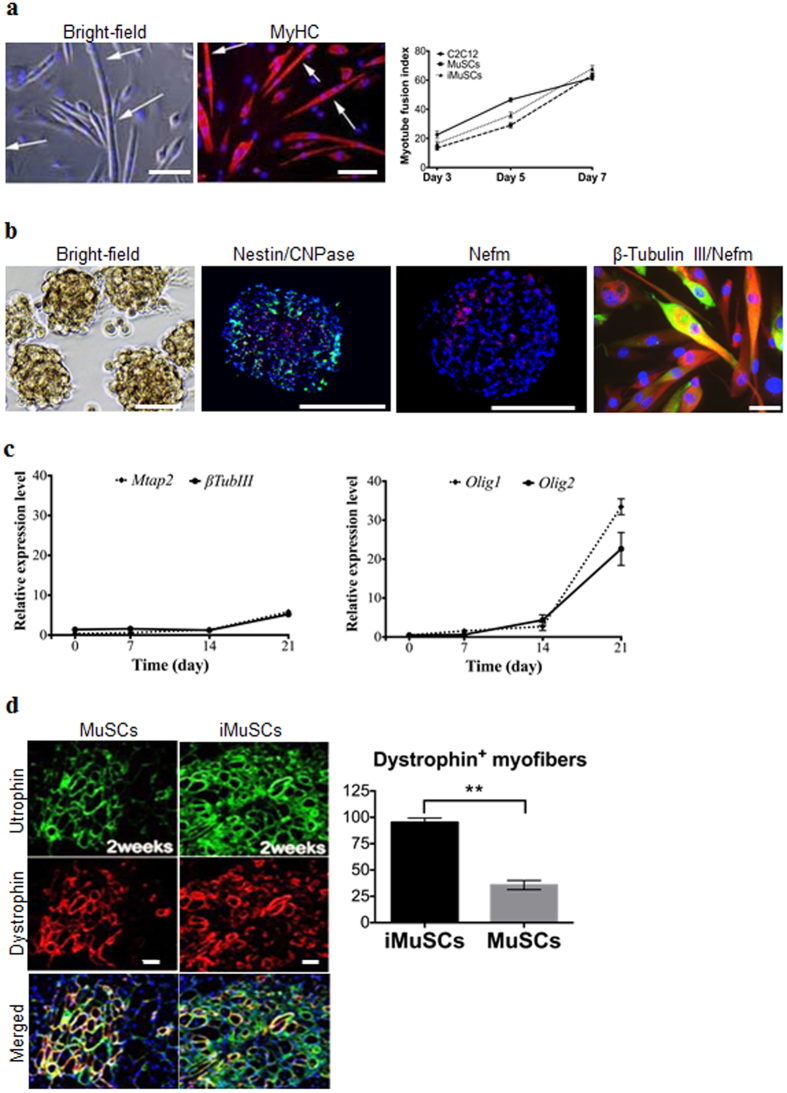
Multiple differentiation and muscle engraftment of iMuSCs. (**a**) Induced myotube formation of iMuSCs. Myotubes expressed MyHC (red). The fusion index was similar to the control C2C12 and MuSCs. (**b**) Representative bright-field picture shows iMuSCs-formed neurospheres floating in suspension. Immunofluorescence staining of cryosectioned neurospheres shows Nestin (green), CNPase (red), and Nefm (red) positive cells. Plated 21 day differentiated neurospheres in ND medium show neural phenotype; β-Tubulin III (red), and Nefm (green). Nuclei were stained with DAPI. Scale bar = 10 and 100 μm. (**c**) Gene expression kinetics of Mtap2 and β-Tubulin III, and Olig1 and Olig2 in the neural differentiating iMuSCs analyzed by qPCR. Data were compared to undifferentiated iMuSCs, and are presented as the mean ±SEM of 5 biological replicates. (**d**) Engraftment of iMuSCs after intramuscular cell implantation. IF staining shows Utrophin+ (green) and Dystrophin+ (red) muscle engraftment of control MuSCs and iMuSCs in mdx/scid mice 2 weeks after cell injection. Scale bar = 100 μm. Quantification of Dystrophin+ myofibers. **P < 0.01.

*In vitro* multipotent differentiation assays showed that iMuSCs were able to fuse with MyHC^+^ (Myosin heavy chain) myotubes in muscle differentiation medium with a similar fusion index as control MuSCs and C2C12 myoblasts (Fig. 2a). The iMuSCs were also capable of differentiating into osteogenic lineages (Supplementary Fig. S2) within osteogenic medium with BMP2. The iMuSCs could also be easily and effectively induced into a neurogenic lineage *via* neurosphere formation once cultured in neural stem cell medium (see Method) for one week (Fig. 2b), whereas the control primary myoblasts and MuSCs showed no sign of forming these structures. The iMuSCs-induced neurospheres exhibited a neural phenotype and expressed Nestin, CNPase and Nefm (Neurofilament) (Fig. 2b). After three weeks, the neurospheres when re-plated in a laminin/polyornithine coated monolayer culture in neural differentiation medium, could differentiate into the three major neural lineages (neurons, astrocytes, and oligodendrocytes) and they expressed Mtap2, β-Tubulin III, Nefm, Nestin and Olig1/2 (Oligodendrocyte transcription factor 1/2) (Fig. 2b,c).

To further investigate the origin of the iMuSCs, we performed *in vivo* intramuscular transplantation studies. Equal numbers of iMuSCs and control MuSCs were injected into the TA muscles of six 6–8 week-old male *mdx/SCID* mice (Jackson Lab, USA). Two and three weeks after cell implantation, we detected Utrophin and Dystrophin (Fig. 2d) expression in the host TA muscles, and observed that the iMuSCs formed larger and more robust Dystrophin^+^ muscle grafts compared to the control MuSCs (Fig. 2d).

**Figure 3:**
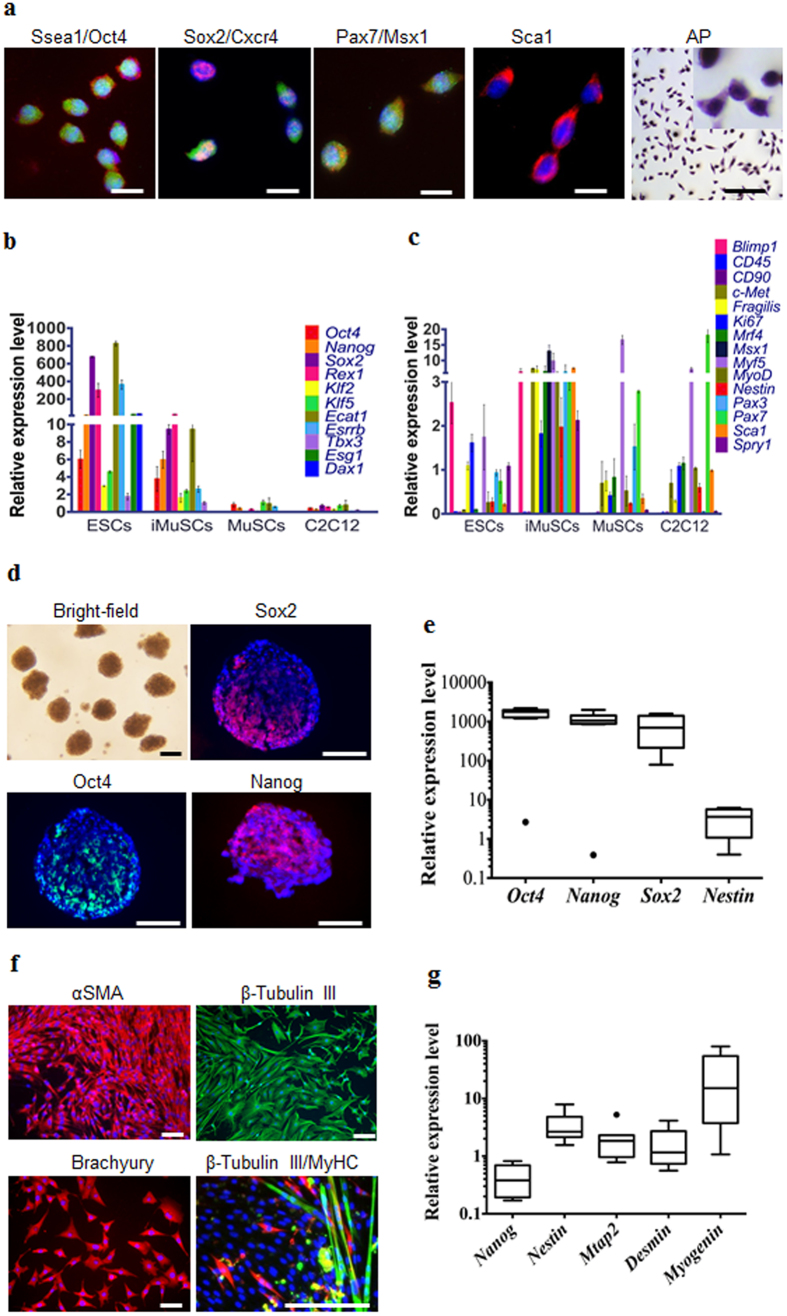
Skeletal muscle injury induced iMuSCs fulfil several *in vitro* criteria of pluripotency. (**a**) Representative immunofluorescent images of iMuSCs expressing Ssea1 (red), Oct4 (green), Sox2 (red), Cxcr4 (green), Pax7 (red), Msx1 (green), Sca1 (red), and alkaline phosphatase. Nuclei were stained with DAPI (blue). Scale bars = 10 and 200 μm. (**b**) qPCR analysis of iMuSCs for pluripotent marker genes and (**c**) myogenic marker genes. Data are represented as the mean ±SEM of 5 biological replicates. (**d**) Bright-field image of formed embryoid bodies (EBs) in suspension, and immunofluorescence staining of cryosectioned EBs. EBs contained cells positive for Sox2 (red), Oct4 (green), and Nanog (red). Nuclei were stained with DAPI (blue). Scale bar = 100 μm. (**e**) qPCR data showed upregulation of Oct4, Nanog, Sox2, and no change of Nestin expression compared to control undifferentiated iMuSCs. (**f**) Immunofluorescence images of differentiated EBs expressing αSMA (red), β-Tubulin III (green), Brachyury (red), and β-Tubulin III β (red) with MyHC (green). Nuclei were stained with DAPI (blue). Scale bar = 100 μm. (**g**) qPCR data of 7 days differentiated EBs show Nestin, Mtap2, Desmin, and Myogenin upregulation, and Nanog downregulation compared to control undifferentiated iMuSCs. All qPCR data was analyzed by the delta-delta Ct method, and are represented as the mean ±SEM of 5 biological replicates.

We also performed quantitative real-time polymerase chain reaction (qPCR) and immunohistochemistry analysis to elucidate the gene and protein expression profile of the iMuSCs and compared these to embryonic stem cells (ESCs) and myogenic stem cells (C2C12 and MuSCs). The iMuSCs expressed Oct4, Ssea1 (Stage-specific embryonic antigen 1), Sox2, Cxcr4, Msx1, Pax7, and Sca1 (Fig. 3a and Supplementary Fig. S3a,b), similar to the ESCs, but at a lower expression level. QPCR analysis revealed that the iMuSCs expressed the majority of pluripotency marker genes, with the exception of *Esg1* and *Dax1* (Fig. 3b); however, unlike the ESCs, the iMuSCs expressed myogenic marker genes and interestingly some of the primordial germ-cell-related markers, e.g. *Blimp1* and *Fragilis*, and did not express other lineage related genes, such as *CD45* or *CD90* (Fig. 3c). Moreover, the iMuSCs were positive for alkaline phosphatase (Fig. 3a). These results indicate that the iMuSCs are similar to, but not identical to the ESCs, since they maintain their myogenic memory (e.g., high expression of myogenic genes when compared to the ESCs, and are easily induced to differentiate into a myogenic lineage *in vitro* and *in vivo*).

**Figure 4:**
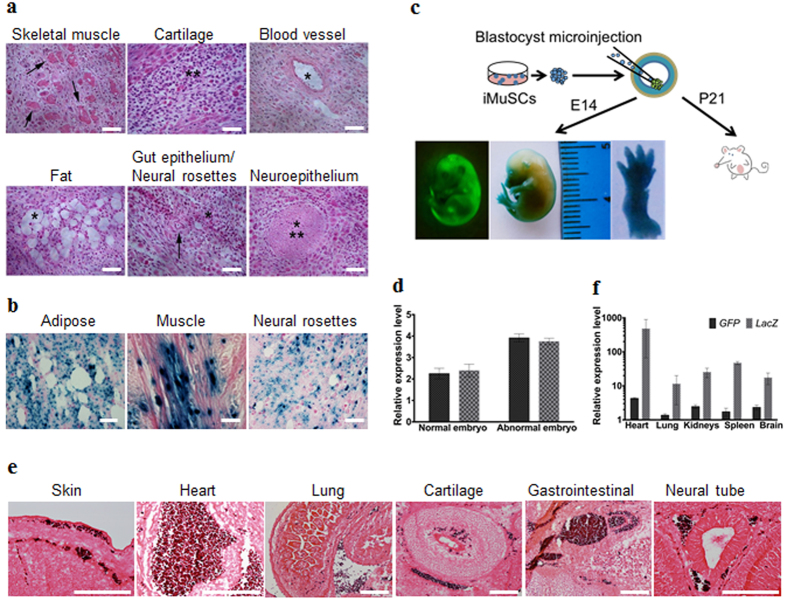
Skeletal muscle injury induced iMuSCs fulfil several *in vivo* criteria of pluripotency. (**a**) Teratoma formation assay of iMuSCs. Hematoxylin and eosin staining showed differentiated structures of all three germ layers: skeletal muscle (mesoderm, arrows), cartilage (mesoderm, asterisks), blood vessel (mesoderm, asterisk), fat (mesoderm, asterisk), gut epithelium (endoderm, asterisk), neural rosettes (ectoderm, arrow), and neuroepithelium (ectoderm, asterisks) within the same sample. (**b**) LacZ staining indicated LacZ pre-labelled iMuSC-differentiated structures of adipose, muscle, and neural rosettes contain β-gal^+^ signals (dark blue dots). Scale bar = 100 μm. (**c**) Contribution of iMuSCs to mouse embryonic development. Embryos at E14 were analyzed by GFP and LacZ staining, (**d**) and by qPCR analysis of *GFP* and *LacZ* marker gene expression in normally and abnormally developed embryos. (**e**) The E14 embryos were sectioned and stained with anti-GFP antibody (dark purple). Cells were counterstained with eosin (pink): skin and under-skin, heart, lung, cartilage, gastrointestinal tract, and neural tube. Scale bar = 200 μm. (**f**) qPCR analysis of *GFP* and *Lac*Z marker gene expression in the born P21 white pups. Data were pulled together from 6 pups.

To clarify the pluripotent potential of the iMuSCs, we performed differentiation assays^6^,^7^ *in vitro* that showed that the iMuSCs were able to form embryoid bodies (EBs) in a petri dish (Fig. 3d,e). After seven days in suspension culture, EBs were expanded and initiated spontaneous differentiation into a variety of ectodermal and mesodermal germ layer derivatives, and after an additional two weeks in culture, attached EBs formed contracting multinucleated myotubes encompassed with neural-like structures (Fig. 3f,g). We further examined the pluripotency of the iMuSCs by teratoma formation *in vivo*. When grafted into *SCID-beige* mice (Jackson Lab, USA) for seven weeks, the iMuSCs formed teratomas (90%, n = 7) containing representative tissues of the three germ layers (Fig. 4a). Histological examination revealed that the iMuSCs differentiated into neural, muscle, and adipose tissues, and epithelium. To verify that the teratomas were formed directly from the implanted cells, the iMuSCs were pre-labelled with β-gal before injection, we detected all three germ layer derivatives in the teratomas contained the β-gal^+^ cells when stained with LacZ (Fig. 4b).

To evaluate whether the iMuSCs could give rise to chimeric mice, a blastocyst injection assay was performed (Fig. 4c). We transferred undifferentiated β-gal^+^ and GFP-pre-labelled iMuSCs as single cells into *BALB/c* (*Jackson Lab, USA*) blastocysts by microinjection following standard procedures^8^. We obtained eight embryos at E14, six of which developed properly and demonstrated the contribution of GFP^+^ iMuSCs to the embryo. A high-to-moderate contribution of β-gal and GFP-expressing cells could be seen in these E14 chimeric embryos (Fig. 4c,d and Supplementary Fig. S4a). Histological analysis confirmed that the iMuSCs contributed to all three germ layers (Fig. 4e and Supplementary Fig. S4b). Offspring derived from the iMuSCs-injected blastocysts were born and developed normally. After repeating this experiment 3 times, we obtained 23 pups, all born with a white coat (Supplementary Table S1). Although their hair did not display iMuSCs germline transmission, immunostaining and qPCR analysis revealed the presence of LacZ^+^ and GFP^+^ iMuSCs in several tissues of the pups, such as skin, muscle, heart, lung, kidneys, spleen, and brain (Fig. 4f and Supplementary Fig. S4c).

## Discussion

The existence of pluripotent-like cells in adult tissues has been a matter of debate for years, since inconsistent results have been reported by various groups^9^,^10^,^11^,^12^,^13^,^14^,^15^; however, no study thus far has proven that such pluripotent stem cells can arise from differentiated somatic tissues. In this study we reveal that cellular reprogramming can be initiated by the strong stimuli that occurs when skeletal muscle is injured; thus, we were able to isolate reprogrammed iMuSCs from the injured skeletal muscle.

Collectively, our findings demonstrate that iMuSCs represent a unique, very sensitive population of cells that possess characteristics (morphology, size, and a gene expression profiles) that differs from all cell types studied so far. IMuSCs not only display several characteristics typical of ESCs (e.g. a large nucleus surrounded by a narrow rim of cytoplasm, high nuclear/cytoplasmic ratio, open chromatin, unstructured nucleoplasm, and diploid number of chromosomes) (Table 1), but also express several pluripotency marker genes while maintaining a high expression level of myogenic genes. Moreover, the most remarkable discovery of this study was that iMuSCs fulfilled several *in vitro* and *in vivo* criteria for pluripotency; however, we could not obtain iMuSCs with germline transmission after blastocyst microinjection. This may be due to the fact that iMuSCs have a lower gene expression profile of the pluripotency markers (e.g., *Oct4, Nanog,* and *Sox2*) and lack *Esg1* and *Dax1* expression when compared to ESCs. It is also plausible that the relatively high expression of *Blimp1*, *Fragilis* and myogenic marker genes by the iMuSCs may contribute to this observation. These results indicate that iMuSCs do not regress completely to pluripotency and possibly hold an epigenetic memory of their myogenic tissue origin. Further manipulation of the iMuSCs, such as the inhibition of DNA methylases or Nanog overexpression, could potentially push the iMuSCs to achieve full pluripotency.

Thus, the key conclusion from our study is that changes in microenvironmental factors, such as skeletal muscle with injuries, can partially reprogram terminally differentiated myogenic cells into a pluripotent-like state.

## Methods

### Animal studies

The animal experiments and all experimental protocols were approved by the Center for Laboratory Animal Medicine and Care at The University of Texas Health Science Center at Houston (protocol No: AWC-13-134). All methods were carried out in accordance with the approved relevant guidelines and regulations. Mouse strains utilized in this study: *C57BL/6J, BALB/c, SCID-beige,* and *mdx/SCID* were purchased from the Jackson Laboratory, USA.

### Cell isolation and maintenance

Mouse iMuSCs were isolated from the injured TA muscles of *C57BL/6J* (3–8-week-old female; Jackson Lab, USA) mice four days after laceration injury, while control MuSCs were isolated from uninjured TA muscles. The iMuSCs were separately cultured in ESGRO Complete PLUS Clonal Grade Medium (Millipore, USA) on 12-well tissue culture plates (Corning, USA) for 3 weeks. The medium was then replaced with normal muscle growth medium [Dulbecco’s Modified Eagle’s Medium (DMEM) supplemented with 20% Fetal Bovine Serum (FBS), 10% Horse Serum (HS), 1% chicken embryo extract (CEE; Accurate Chemical Co., UK), and 1% Penicillin-Streptomycin antibiotics; unless otherwise mentioned, all from Gibco, USA] and iMuSCs were further cultured and expanded on collagen type IV-coated flasks at 5% CO_2_ at 37 °C. C2C12 primary mouse myoblasts (purchased from ATTC, USA) and MuSCs were used as controls and were cultured on collagen type IV-coated flasks in growth medium in 5% CO_2_ at 37 °C. Characterization of iMuSCs was performed by applying standard *in vitro* and *in vivo* assays.

### Generation and analysis of chimeric mice

Undifferentiated single cells of β-gal- and GFP-pre-labelled iMuSCs were transferred into blastocysts of *BALB/c* mice (Jackson Lab, USA) by microinjection following standard procedures^8^. Pregnant mice were sacrificed and E14 embryos were collected and dehydrated in a serial dilution of 30, 20 and 10% sucrose and then fixed in 4% paraformaldehyde (Sigma, USA) and embedded in paraffin. Sections were stained with anti-GFP antibody, counterstained with eosin and visualized by fluorescent microscopy (Nikon).

### Teratoma formation assay

IMuSCs were suspended at 1 − 2 × 10^6^ cells/ml in Phosphate Buffered Saline (PBS). *SCID-beige* mice (Jackson Lab, USA) were anesthetized with diethyl ether and injected with 500 μl of the cell suspension subcutaneously into the dorsal flank. Seven weeks after the cell injection, tumours were surgically dissected from the mice. Samples were weighed, fixed in 4% formaldehyde, and embedded in paraffin. Sections were stained with hematoxylin and eosin.

### *In vitro* differentiation

*In vitro* embryoid body (EB) formation of iMuSCs was induced by applying the routinely used ‘hanging-drop’ technique^6^,^16^. Single cell suspensions of iMuSCs (3.75 × 10^4^ cells/ml) were placed as 20 μl micro-drops of ESGRO Complete PLUS Clonal Grade Medium (Millipore, USA) on the lids of 100 mm dishes. Bottom plates of the dishes were filled with sterile PBS to avoid desiccation of samples. Lids were then placed on the bottom plates for 4 days to achieve hanging drop cultures. During this time, single iMuSCs formed EBs, which were harvested and further cultured in suspension for 3 additional days. For spontaneous *in vitro* differentiation, EBs were cultured on collagen type IV-coated 24-well plates in EB Medium [DMEM supplemented with 20% FBS, 2 mM Glutamax, 1% non-essential amino acids, and 1% antibiotics; all from Gibco (USA)] for 2 weeks with medium changed every other day. Additionally, multiple differentiations of iMuSCs were assessed by myogenic and osteogenic differentiation assays following standard protocols^5^,^17^,^18^.

### Singe cell migration

Target cell populations and control cells (MuSCs and C2C12 cells) were plated separately on collagen type IV-coated 6-well plates, 10,000 cells/well, in growth medium, 48 hours prior to the time-lapse experiment. Time lapse images were acquired with an Andor IXon3 885 EMCCD camera (Andor, USA) on an Olympus IX-81 (Olympus, USA) microscope fitted with a microscope enclosure (Precision Plastics, USA), and images of single cell migration were taken for six hours at three minute intervals; three different fields/well were chosen for the recording. Proper environmental conditions were maintained in a micro incubator at 37 °C with 5% CO_2_. A series of images were analyzed using NIH ImageJ analysis software to track the centroid positions (x, y) of cell nuclei (which were assumed to be the representations of cell-bodies). Migration paths were plotted and analyzed by the Chemotaxis and Migration Tool v2.0 from Ibidi. Net translocation distance was measured as the distance between the starting point and the end point of cells after six hours. Migration speed was calculated as total length of the migration path during the six-hour period.

### Immunohistochemistry

Samples were fixed with 4% paraformaldehyde (Sigma, USA) for 20 minutes at room temperature. After permeabilization with 0.2% Triton X-100 (Sigma, USA) for 30 minutes, nonspecific binding of antibodies was blocked for one hour with 10% BSA and 5% HS (Sigma, USA) in PBS, at room temperature. The primary antibodies (Supplementary Table 2) were applied overnight at 4 °C. After the overnight incubation, the cells were incubated for one hour at room temperature with the appropriate fluorescence-conjugated secondary antibodies (Extended Data Table 2). The nuclei were revealed using 4′, 6′-diamidino-2-phenylindole (DAPI) staining (Sigma, USA), and fluorescent microscopy (Nikon) was used to visualize the results. Quantitative image analysis was performed using the NIH ImageJ Software.

### Karyotype analysis

Karyotypes were determined using conventional G-Banding analysis at the Texas Children’s Hospital Cytogenetic Core Lab, Houston, TX, USA.

### Population doubling assay and histochemical staining for alkaline phosphatase

To estimate the growth of cultures, a standard protocol was followed for population doubling assays. Briefly, 5000 iMuSCs were plated on collagen type IV-coated 6-well plates in growth medium for four days, and every day the well content was collected and counted. The approximate population doubling time (PDT) was determined as: 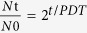, where, t = time period (24 h), N_0_ = initial cell number, N_t_ = number of cells at the time period.

Histological staining for alkaline phosphatase activity was carried out using a commercially available kit (Sigma-Aldrich, USA) following the manufacturer’s instructions.

### Quantitative real-time PCR

Total RNA was isolated using the RNeasy Plus Mini Kit (Quiagen, USA), and cDNA was synthesized from 1 μg of RNA *via* a High Capacity RNA-to-cDNA Kit (Life Technologies, USA) following the manufacturer’s instructions. Gene expression was analyzed by quantitative real-time PCR (qPCR) using MyiQ real time PCR System (Bio-Rad, USA). The applied primers (Supplementary Table 3) were designed by Oligo software (Oligo Perfect Designer, Invitrogen, USA). Reactions were measured in duplicates using custom 2× Syber Green Master Mix based on the hot-start Jumpstart Taq DNA Polymerase enzyme (Sigma, USA). The amplification was done for 40 cycles (95 °C 20 sec, 60 °C 20 sec, 72 °C 40 sec). To verify the PCR products, melting curves and negative controls were carried out in each reaction. Relative quantification of mRNA was determined by the ΔΔCt method (2^−ΔΔCt^ formula)^19^ by using the expression profile of the corresponding control samples as reference.

### Data processing and statistical analysis

Prism 6.0 (GraphPad Software, USA) was used for data plotting, non-linear regression and statistical analysis. Data are given as mean ± S.E.M. Comparisons between two groups were performed by using Student’s t-test assuming two-tailed distribution, and unequal variances. For multiple comparisons, ANOVA or Kruskal-Wallis test was applied. Statistical significance was considered at p < 0.05.

## Additional Information

**How to cite this article**: Vojnits, K. *et al.* Characterization of an Injury Induced Population of Muscle-Derived Stem Cell-Like Cells. *Sci. Rep.* **5**, 17355; doi: 10.1038/srep17355 (2015).

## Supplementary Material

Supplementary Information

## Figures and Tables

**Table 1:** Shared characteristics and differences between ESCs and iMuSCs.

*In vitro* criteria	Mouse ESCs	References	iMuSCs
Undifferentiated morphology	round shaped cells with high nuclear/cytoplasm ratio	20, 21, 22	round shaped cells with high nuclear/cytoplasm ratio
	can grow as single cells in monolayer		can grow as single cells in monolayer
Visco-elastic properties	viscous, which is changing during differentiation	23	?
Self-renewal	yes	20, 21, 22, 23	yes
Cell surface marker expression	SSEA-1, c-kit, LIFR	24	SSEA-1, Sca-1, CXCR-4, CD-34
Alkaline phosphatase activity	high level	22	high level
Telomerase activity	high mTRT expression	25	?
“Stemness” gene expression	Oct-3/4, Nanog, Sox-2, Klf-2, Klf-5, Klf-4, GDF-3, Rex-1, Ecat-1, Stat-3, Fox-D3, Vasa, Shall-4, Dax-1, Esrrb, Esg-1, Tbx-3, Tcl-1, Rif-2, Nac-1, Zfp-281, Dppa-3	21,24	Oct-3/4, Nanog, Sox-2, Rex-1, Klf-2, Klf-5, Ecat-1, Esrrb, Tbx-3, Blimp-1, c-Met, Fragilis, Ki67, Mrf-4, Msx-1, Myf-5, MyoD, Nesitn, Pax-3, Pax-7, Spry-1
Genetic/Epigenetic properties	demethylation of Oct-3/4, Rex-1, Nanog promoters	21,26	?
Genetic stability	stable, maintain normal karyotype	20	stable, normal diploid; at high passages trisomy for chromosome 5
Bivalent chromatin structure	yes	26,27	?
Embryoid body formation	yes	20,21	yes
Ectoderm differentiation	β-Tubulin-III, Mtap-2, Ncam-1, Nestin, Pax-6, Sox-1, Gfap, Olig-1, Olig-2	28, 29, 30	β-Tubulin-III, Mtap-2, Nestin, Olig-1, Olig-2
Endoderm differentiation	Bmp-4, Cytokeratins, Hnf-4, Somatostatin	31, 32, 33	Afp
Mesoderm differentiation	α-Cardiac actin, Gata-4, Brachyury, Myh-6/7, Nppa, Myf-5, MyoD	34,35	Brachyury, α-Smooth muscle actin, Myh-6/7, Desmin, Myogenin
*In vivo* criteria			
Teratoma formation	yes	20,21	yes
Chimera formation	yes	21,36	yes/no

Abbreviations: ESC, embryonic stem cells; iMuSCs, injured muscle-derived stem cell-like cells.
