# Supplementary material for: Characterization of an Injury Induced Population of Muscle-Derived Stem Cell-Like Cells
Source: Sci Rep. 2015 Nov 27;5:17355. doi: 10.1038/srep17355 (PMC4661568; doi:10.1038/srep17355)
Supplement: Supplementary Information [file srep17355-s1.doc]

**Characterization of an Injury Induced Population of Muscle-Derived Stem Cell-Like Cells**

**Authors:** Kinga Vojnits ^1,2^, HaiYing Pan ^1,2^ , Xiadong Mu ^3^ , Yong Li* ^1,2^

**Affiliations:**

^1^Department of Pediatric Surgery, University of Texas Medical School at Houston, TX 77030, USA.

^2^Center for Stem Cell and Regenerative Medicine, University of Texas Health Science Center at Houston (UTHealth), TX 77030, USA.

^3^Stem Cell Research Center, University of Pittsburgh, Medical School, Pittsburgh, PA 15213, USA.

*Correspondence to: Yong Li, M.D. Ph.D. ([Yong.Li.1@uth.tmc.edu](mailto:Yong.Li.1@uth.tmc.edu))

**Figures**


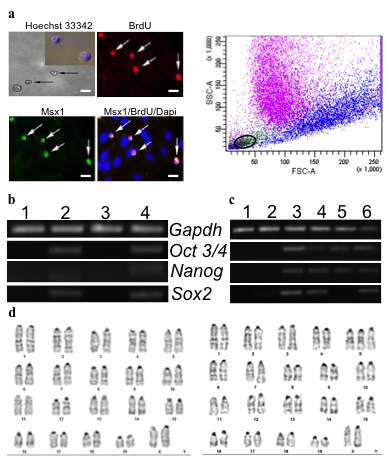


**Supplementary Figure 1: Characterization of iMuSCs**

**a,** Bright field image of fresh isolated iMuSCs, which were Hoechst 33342 positive and incorporated BrdU (red) with Msx1 (green) expression. Nuclei were stained with DAPI (blue). Scale bar = 10μm. Sca1^+^ and CD34^+^ small sized iMuSCs were sorted from the mixed cell culture by FACS. **b,** Expression of the pluripotency marker genes in the whole biopsied TA tissues. Samples: old control uninjured TA (line 1), old injured TA (line 2), young uninjured TA (line 3), young injured TA (line 4). **c,** Expression of the pluripotency marker genes in the cells. Samples: C2C12 (line 1), MuSCs (line 2), ESCs (line 3), iMuSCs passage 9 (line 4), fresh isolated iMuSCs exp1 (line 5), fresh isolated iMuSCs exp2 (line 6). **d,** Karyotype analysis of cultured iMuSCs passage 13 (left panel) and passage 33 (right panel).


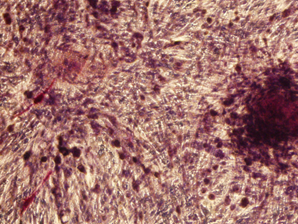


**Supplementary Figure 2: Osteogenic differentiation of iMuSCs.**

IMuSCs were induced to osteogenic differentiation and stained by ALP kit. Scale bar = 100μm.


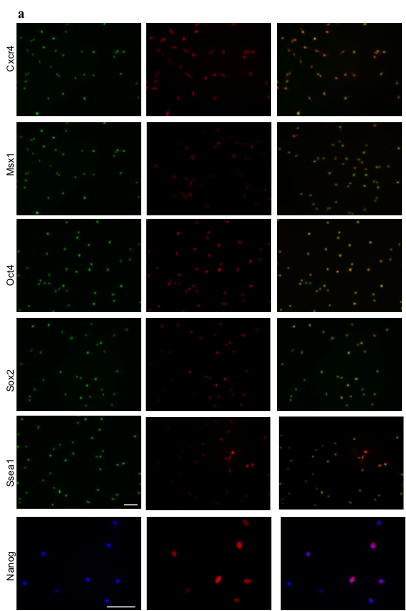


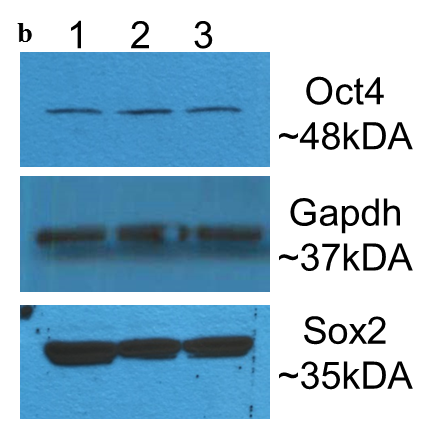


**Supplementary Figure 3: Protein expression profile of iMuSCs.**

**a,** Representative immunofluorescence images of iMuSCs expressing Cxcr4, Msx1, Oct4, Sox2, Ssea1, and Nanog. Nuclei were stained with DAPI (green, blue). Scale bar = 100μm. **b,** Oct4, Sox2, and Gapdh expression of iMuSCs passage 9, 11, 13 (line 1, 2, 3) analysed by Western blot.


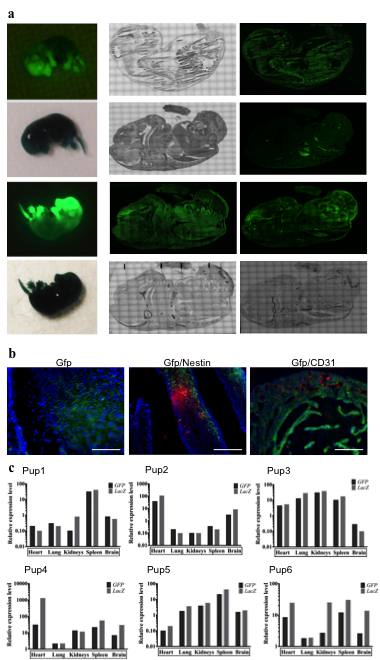


**Supplementary Figure 4: Contribution of iMuSCs to mouse embryonic development.**

**a,** Embryos at E14 were analysed by GFP and LacZ staining. **b,** The E14 embryos were sectioned and stained with GFP (green), Nestin (red), and CD31 (red). Nuclei were stained with DAPI (blue). Scale bar = 100μm. **c,** qpCR analysis of *GFP* and *LacZ* marker gene expression in 6 born P21 white pups.

**Tables**

**Supplementary Table 1: Blastocyst microinjection.**

| **Experiment** | **Treatment** | **Nb. of injected cells / nb. of blastocysts** | **Nb. of GFP+ embryos** | **Nb. of pups** | **Coat colour** |
| --- | --- | --- | --- | --- | --- |
| **1** | MuSC Medium | 12/11cells | 8/6 ok | - | - |
|  |  | 12/11cells | - | 6 | White |
| **2** | ESC Medium | 14/18 cells | - | 4/1 died | White |
|  |  | 12/11 cells | - | 3 | White |
| **3** | MuSC Medium | 16/11 cells | - | 10 | White |

**Abbreviations:** MuSC, muscle stem cell; ESC, embryonic stem cell.

**Supplementary Table 2: Primary and secondary antibodies.**

| **Primary antibodies** | **Host** | **Dilution** | **Manufacturer** | **Secondary antibodies** |
| --- | --- | --- | --- | --- |
| Ssea1 | Mouse monoclonal IgM | 1:150 | Santa Cruz | Goat anti-mouse AlexaFluor 594 |
| Oct4 | Rabbit polyclonal IgG | 1:200 | Abcam | Goat anti-rabbit AlexaFluor 488 |
| Sox2 | Mouse monoclonal IgG1 | 1:200 | Sigma | Goat anti-mouse AlexaFluor 594 |
| Cxcr4 | Rabbit polyclonal IgG | 1:150 | Santa Cruz | Goat anti-rabbit AlexaFluor 488 |
| Pax7 | Mouse monoclonal IgG1 | 1:200 | DSHB | Goat anti-mouse AlexaFluor 594 |
| Msx1 | Rabbit plyclonal IgG | 1:150 | Abcam | Goat anti-rabbit AlexaFluor 488 |
| Sca1 | Rat IgG2a | 1:250 | BD Biosciences | Goat anti-rat AlexaFluor 594 |
| Nanog | Mouse monoclonal IgG1 | 1:150 | Santa Cruz | Goat anti-mouse AlexaFluor 647 |
| α-Smooth muscle actin | Mouse monoclonal IgG2a | 1:500 | Sigma | Goat anti-mouse AlexaFluor 594 |
| β-Tubulin III | Rabbit plyclonal IgG | 1:500 | Abcam | Goat anti-rabbit AlexaFluor 488/594 |
| Brachyury | Rabbit polyclonal IgG | 1:200 | Sigma | Goat anti-rabbit AlexaFluor 594 |
| Myosin heavy chain | Mouse monoclonal IgG1 | 1:200 | Sigma | Goat anti-mouse AlexaFluor 488 |

**Supplementary** Table 3: PCR primers.

| **Gene** | **5’Primer** | **3’Primer** |
| --- | --- | --- |
| **β-Actin** | GGCTGTATTCCCCTCCATCG | CCAGTTGGTAACAATGCCATGT |
| **β-Catenin** | GCGTGGACAATGGCTACTCAAG | TATTAACTACCACCTGGTCCTC |
| **Blimp1** | CACACAGGAGAGAAGCCACA | TTGTGACACTGGGCACACTT |
| **c-Met** | CTCCTGACATCCATCTCCACC | TCTTCTTTCCTGTGACAACCAA |
| **CD34** | AAGGCTGGGTGAAGACCCTTA | TGAATGGCCGTTTCTGGAAGT |
| **CD45** | CCATATGTTTGCTTTCCTTCTCC | TGGTGACTTTTGGCAGATGA |
| **CD90** | CGCTCTCCTGCTAACAGTCTT | CAGGCTGAACTCGTACTGGA |
| **Dax1** | TGCTGCGGTCCAGGCCATCAAGAG | GGGCACTGTTCAGTTCAGCGGATC |
| **Desmin** | GTGGATGCAGCCACTCTAGC | TTAGCCGCGATGGTCTCATAC |
| **E-Cadherin** | AATGGCGGCAATGCAATCCCAAGA | TGCCACAGACCGATTGTGGAGATA |
| **Ecat1** | TGTGGGGCCCTGAAAGGCGAGCTGAGAT | ATGGGCCGCCATACGACGACGCTCAACT |
| **Endogenous Nanog** | CAGGTGTTTGAGGGTAGCTC | CGGTTCATCATGGTACAGTC |
| **Endogenous Oct3/4** | TCTTTCCACCAGGCCCCCGGCTC | TGCGGGCGGACATGGGGAGATCC |
| **Endogenous Sox2** | TAGAGCTAGACTCCGGGCGATGA | TTGCCTTAAACAAGACCACGAAA |
| **Esg1** | GAAGTCTGGTTCCTTGGCAGGATG | ACTCGATACACTGGCCTAGC |
| **Esrrb** | TTTCTGGAACCCATGGAGAG | AGCCAGCACCTCCTTCTACA |
| **Fragilis** | AACATGCCCAGAGAGGTGTC | CTTAGCAGTGGAGGCGTAGG |
| **Gapdh** | AACTTTGGCATTGTGGAAGG | GGATGCAGGGATGATGTTCT |
| **GFP** | AAGTTCATCTGCACCACCG | TCCTTGGAAGAAGATGGTGCG |
| **Ki67** | GCAGGTTAGCACTGTTATGAAAAC | GGGCCTTGGCTGTTTTACATT |
| **Klf2** | GCCTGTGGGTTCGCTATAAA | AAGGAATGGTCAGCCACATC |
| **Klf5** | CAAGCCGTTCCAGTGCAT | GTCTGCGGTTTAAAGGATGG |
| **LacZ** | TTCACTGGCCGTCGTTTTACAACGTCGTGA | ATGTGAGCGAGTAACAACCCGTCGGATTCT |
| **M-Cadherin** | TCGGGCTGCTTGCCCAGAG | CCCTGGATGCTGTAGATGACACTGC |
| **Mrf4** | GCACCGGCTGGATCAGCAAGAG | CTGAGGCATCCACGTTTCTCC |
| **Msx1** | TCCCTCAAGCTGCCAGAAGA | TTGTGCTTGCGTAGGGTG |
| **Mtap2** | ACACCCCGAACCAGGAGGA | GCGTTGGACGTGCCCTTCT |
| **Myf5** | CCTGTCTGGTCCCGAAAGAAC | TAGACGTGATCCGATCCACAAT |
| **MyoD** | CCACTCCGGGACATAGACTTG | AAAAGCGCAGGTCTGGTGAG |
| **Myogenin** | GGTGTGTAAGAGGAAGTCTGTG | TAGGCGCTCAATGTACTGGAT |
| **N-Cadherin** | GGCGTCTGTGGAGGCTTCTGGTGAA | GTGATGACGGCTGTGGCTGTGTTTGA |
| **Nestin** | CTGGAAGGTGGGCAGCAACT | ATTAGGCAAGGGGGAAGAGAAGGATG |
| **Olig1** | ACGTCGTAGCGCAGGCTTAT | CGCCCAACTCCGCTTACTT |
| **Olig2** | GGGAGGCGCCATTGTACA | GTGCAGGCAGGAAGTTCCA |
| **Pax3** | TCCGAGCACTGTACACCAAAGC | CGATGGAGGCACAAAGCTGTC |
| **Pax7** | TCTCCAAGATTCTGTGCCGAT | CGGGGTTCTCTCTCTTATACTCC |
| **Rex1** | ACGAGTGGCAGTTTCTTCTTGGGA | TATGACTCACTTCCAGGGGGCACT |
| **Sca1** | AGGAGGCAGCAGTTATTGTGG | CGTTGACCTTAGTACCCAGGA |
| **Spry1** | CTTTGTGCCTACCCTGCTTGCTCTGCTACC | AGGGCGGTGGGTCCAGTCGTAACAGC |
| **Tbx3** | AGGAGCGTGTCTTGCAGGTT | GCCATTACCTCCCCAATTTT |
| **Total Nanog** | AGGGTCTGCTACTGAGATGCTCTG | CAACCACTGGTTTTTCTGCCACCG |
| **Total Oct3/4** | CTGAGGGCCAGGCAGGAGCACGAG | CTGTAGGGAGGGCTTCGGGCACTT |
| **Total Sox2** | GGTTACCTCTTCCTCCCACTCCAG | TCACATGTGCGACAGGGGCAG |
| **β-TubulinIII** | AGACAACTTCGTTTTCGGTCAGT | CCTTTAGCCCAGTTGTTGCCT |
